# Trauma, Posttraumatic Stress Disorder, and Treatment Among Middle-Aged And Older Women

**DOI:** 10.1093/geroni/igab046.1587

**Published:** 2021-12-17

**Authors:** Laura Sampson, Shaili Jha, Andrew Ratanatharathorn, Andrea L Roberts, Laura D Kubzansky, Eric B Rimm, Karestan C Koenen

**Affiliations:** 1 Harvard T.H. Chan School of Public Health, Boston, Massachusetts, United States; 2 Harvard T.H. Chan School of Public Health, Harvard T.H. Chan School of Public Health, Massachusetts, United States

## Abstract

Posttraumatic stress disorder (PTSD) is twice as prevalent in women as in men, and is an established risk factor for chronic disease, but few studies have comprehensively assessed lifetime PTSD in middle-aged and older civilian women. We surveyed 33,328 women aged 54-74 from the Nurses’ Health Study II from August 2018 to January 2020 to understand trauma exposure, PTSD based on the Diagnostic and Statistical Manual of Mental Disorders Version 5, and trauma-related treatment use. The majority (82.2%) of women reported one or more lifetime traumas. 10.5% of those with trauma had lifetime PTSD and 1.5% had past-month PTSD. The most common trauma types were sudden or unexpected death of a loved one (44.9%) and interpersonal or sexual violence (43.5%). Almost 30% experienced occupational (nursing-related) trauma. Interpersonal or sexual violence event types explained the largest proportion of PTSD cases (33.6%) out of seven categories of events assessed. Only 25% of women with trauma ever accessed trauma-related treatment, but this proportion was higher (66.4%) among those with diagnosable PTSD, and among those with current depression (35.9%). Treatment was most common among women who experienced interpersonal/sexual violence and lowest among those with occupational trauma, but treatment satisfaction did not vary by worst trauma type. Psychotherapy was the most common type of treatment. These results demonstrate that trauma is nearly universal in middle-aged to older women, which has important implications for their long-term health and well-being—particularly in the era of COVID-19 which is likely to produce additional trauma in this population.

